# Gateway to Syntax: On the Neural Origins of the Left Anterior Negativity and Their Functional Implications

**DOI:** 10.1162/NOL.a.227

**Published:** 2026-03-26

**Authors:** Manuel Martin-Loeches, Werner Sommer, Laura Jiménez-Ortega, Javier Espuny, Miguel Rubianes, Pilar Casado

**Affiliations:** Center UCM-ISCIII for Human Evolution and Behavior, Madrid, Spain; Department of Psychobiology and Methods in Behavioral Sciences, Universidad Complutense de Madrid, Spain; Department of Psychology, Humboldt Universität zu Berlin, Germany; Life Sciences Imaging Center, Hong Kong Baptist University, Hong Kong, China; Faculty of Education, National University of Malaysia, Kuala Lumpur, Malaysia; Faculty of Health Sciences, UNIE University, Madrid, Spain

**Keywords:** syntax processing, language, left anterior negativity (LAN), neural sources

## Abstract

The neural origins of the Left Anterior Negativity (LAN) component of the Event-Related brain Potentials (ERP) have never been directly probed although this information is of the highest interest for a comprehensive view of the neural foundation of language. The LAN emerges specifically after morphosyntactic violations and is affected by both linguistic and extralinguistic, non-syntactic information. Here, we explored the neural sources of the LAN by analyzing data from three previously published ERP data sets obtained from canonical morphosyntactic violation conditions. The neuroelectric source analyses were based on LAN data from *N* = 76 participants and comprised two distributed source algorithms: sLORETA (standardized low-resolution brain electromagnetic tomography), and CLARA (classical LORETA analysis recursively applied), and a discrete dipole model (BESA, brain electrical source analysis). The results indicate that the most acceptable candidate as primary neural source of the LAN is the left frontal operculum (LFO), though the right FO might also be implicated. Considering its location, functions and connections, we speculate that the FO may be monitoring articulatory (morphological or morphophonological) predictions during language comprehension. The direct links between the FO and the anterior temporal pole might also account for nonlinguistic influences on the LAN.

## INTRODUCTION

In the scientific study of human language, the role of Event-Related brain potentials (ERP), a technique based on the electroencephalogram (EEG) has been critical, given that it provides temporally fine-grained information, at the millisecond level, about the unfolding of linguistic processes. ERP findings complement information from anatomically more accurate techniques, such as functional Magnetic Resonance Imaging (fMRI) and Positron Emission Tomography. For comprehensive neurocognitive theories it is important to combine the various bodies of evidence from different methods into consistent accounts.

Traditionally, language has been partitioned into different domains. Among these, phonology, syntax and semantics stand out, with the latter two probably being the most studied and established through the ERP method (e.g., Sun & Luo, 2024). ERP wave shapes contain several components with distinct functional significance, of which three have been described in the literature as relatively specific for syntactic processing: the Early Left Anterior Negativity (ELAN), the Left Anterior Negativity (LAN), and the P600. The ELAN is a negative deflection at left frontal electrodes observed at 100–300 ms after stimulus onset, which has been distinguished from the later LAN effects in the 300–500 latency window. According to a highly influential model (Friederici, 1995, 2002, 2017), the ELAN may index difficulties during an initial stage of phrase-structure building during which word category information is identified. Later on, the LAN would index difficulties during morphosyntactic information processing and integration. Indeed, the ELAN is most reliably elicited by word category violations, whereas the later LAN is elicited by a broader range of conditions, especially morphosyntactic violations of number, case, gender, or tense (Palolahti et al., 2005; Steinhauer & Drury, 2012). In turn, the P600 is a centro-parietal positive component peaking around 600 ms after the onset of an outstanding syntactic event (Kaan et al., 2000; Osterhout & Holcomb, 1992). The P600 has been interpreted as a general marker for processing, reanalysis and integration of sentence structure (Aurnhammer et al., 2023; Brouwer et al., 2012, 2017; Friederici, 2011; Steinhauer & Connolly, 2008); in contrast, the functional interpretations of the ELAN and LAN components appear to be more controversial, as outlined in the following.

At variance with the P600 component the ELAN and LAN components are most consistently elicited by syntactic and morphosyntactic violations and might therefore index problems during syntactic processing. In his Memory, Unification, Control model, Hagoort (2013) suggested that anterior negativities result when a syntactic element cannot be united with any other element in the current parse, because of violations of word-category or morphosyntactic information. Alternatively, it has been proposed that anterior negativities might more broadly index working memory operations involved in the processing of syntactic material (Fiebach et al., 2001; King & Kutas, 1995; Kluender & Kutas, 1993). Even though, when measured in the same study, anterior negativities due to working memory demands seem to be more centrally distributed than the typical LAN due to morphosyntactic violations (Martín-Loeches et al., 2005). Indeed, at least the LAN is rather variable across studies both in its scalp topography and even in whether it is present or not (Ciaccio et al., 2023; Grevisse et al., 2023; Tanner & Van Hell, 2014).

Several studies using the LAN as a tool in psycholinguistic research have shown that syntactic processing may not be as encapsulated as traditionally held (e.g., Fodor, 1983; Hauser et al., 2002), and that semantics and even extralinguistic variables may interact and interplay with syntax already at early processing stages. Thus, several recent studies have reported effects of emotions on syntactic processing, as reflected in the LAN. When emotional information concurs with linguistic violations the LAN may be reduced or enhanced in amplitude (Espuny et al., 2018; Jiménez-Ortega et al., 2017; Martín-Loeches et al., 2012; Vieitez et al., 2024), and may even vanish or be replaced by the N400, a more centrally distributed ERP component related to semantic processing (Jiménez-Ortega et al., 2020; Martín-Loeches et al., 2012). Further, social variables such as the presence of a confederate (Hinchcliffe et al., 2020) or the inclusion of self-related information (Rubianes et al., 2024) may alter the amplitude or even the occurrence of the LAN. Overall, the influence of extralinguistic cues on syntax processing seems undisputable, a conclusion that is at least in part based on LAN findings.

Considering the influential contributions of anterior negativities to models of psycholinguistics (e.g., Friederici, 2017; Hagoort, 2013; Townsend & Bever, 2001), and in view of the large amount of available data on factors influencing them, an up-to-date neurofunctional account of these brain electrical fluctuations appears necessary that can be integrated with current knowledge on the neurophysiological mechanisms of language processing at large (e.g., Aliko et al., 2023; Fedorenko et al., 2024; Hickok, 2022; Woolnough et al., 2023). A necessary step in this endeavor is the elucidation of their neural origins.

Indeed, the situation about the neuroelectric sources of the frontal negativities is somewhat complex, as whether the ELAN and LAN are different components related to functionally distinct language processes, or whether they reflect the same process that merely varies in onset latency, is a matter of debate (Swaab et al., 2011; Turco & Houghton, 2022). Indeed, several studies have not been able to dissociate these LAN components with standard linguistic manipulations. For example, Hinojosa et al. (2003) used phrase structure and morphosyntactic violations in Spanish; both violations appeared to yield LAN between 250 and 400 ms. Finally, and importantly, according to the model, the two responses should originate in anatomically different neural loci. Following most recent versions of the model (Friederici, 2017, see specifically pp. 37–43), the ELAN is supposed to originate in the deep frontal operculum (FO) contiguous to the anterior insula and the anterior portions of the superior temporal gyrus (STG), of both hemispheres, with both areas being connected within each hemisphere through the uncinate fasciculus. In turn, the LAN is considered to be generated mainly within the traditional Broca’s area, left BA44, which is tightly connected to the posterior portion of the STG through the arcuate fasciculus. In this regard, although the *pars opercularis* of the inferior frontal gyrus -IFG- (roughly corresponding to BA44) and IFG *pars triangularis* (roughly corresponding to BA45) have been proposed as forming Broca’s area together with FO, connectivity-based parcellation studies have shown that the surface regions BA44/45 are segregated from FO (Anwander et al., 2007).

Interestingly, when reviewing the literature on the neural origins of the anterior negativities (either the ELAN or the subsequent LAN), a striking finding is that no study has directly afforded the location of the neural generators for the LAN. Indeed, as explicitly mentioned by Friederici (2002, 2017), whereas the neural generators of the ELAN have been verified by dipole localization, the neural sources of the LAN have been associated with the BA44 by its function rather than by direct generator localization. In other words, the neural generators of the LAN have never been directly addressed; the assumption that it originates in BA44 is based on fMRI data suggesting that this region supports the functions presumably reflected by the LAN. Moreover, the neural origins of the ELAN are far from being firmly established. The suggestion that the FO contributes to the ELAN is mostly based on studies with small participant samples, such as Friederici et al. (2000), Gross et al. (1998), and Knösche et al. (1999), with *N* = 5, 10, and 6, respectively; alternatively, on studies of the *syntactic mismatch response*, a negativity that is obtained from isolated pairs of words that can match or mismatch grammatically (e.g., “we come” vs. “we comes”; Herrmann et al., 2009; Pulvermüller & Shtyrov, 2003), which is different from the design typically giving rise to the LAN or ELAN. Further, several of these localization studies (e.g., Gross et al., 1998; Knösche et al., 1999) used limited methods such as the current source density analysis or have explicitly seeded bilateral frontal equivalent dipoles using the results of an fMRI study as a prior (Friederici et al., 2000), neglecting alternative options. To date, the only study without most of these limitations is by Herrmann et al. (2011), using a distributed source model (sLORETA), which accounts for activations over the whole cortical surface without prior assumptions about source locations, while using a relatively large sample size of *N* = 24. According to their analyses Herrmann et al. (2011) concluded that the ELAN originates in bilateral anterior portions of the STG in BA22, a result that is consistent with the findings of Friederici et al. (2000), Gross et al. (1998), and Knösche et al. (1999).

All the studies directly affording the neural generators of the ELAN have used data from magnetoencephalography (MEG), hence, they have specifically investigated the ELANm, although most studies on ELAN and LAN in the literature have used EEG. However, as Herrmann et al. (2011, p. 631) suggested, “EEG and MEG may measure different parts of the same phenomenon, due to the orientation of the dipoles”, and concluded that “with MEG alone, the question about a frontal source of the ELAN/ELANm component is not conclusive.” Accordingly, while the role of the STG in the generation of the ELANm appears to be acknowledged the involvement of the inferior frontal cortex in generating the electrical ELAN remains less clear. Finally, the ELAN has been shown to be absent in patients with selective lesions in the left inferior frontal cortex or in the anterior temporal lobe, but not if the lesion affects the basal ganglia, such as the caudate nucleus (Friederici et al., 1999; for a review see Friederici & Kotz, 2003), providing support for a frontal as well as a temporal contribution to this component.

In the present study we aimed to elucidate the neural origins of the later LAN component in the ERP, which is necessary to integrate the functional properties of the anterior negativities within neurocognitive models of language. To achieve this goal, we analyzed a large sample of *N* = 76 participants, using a comprehensive approach to source modelling. The data analyzed here were extracted from previous studies from our lab in which the LAN had been obtained under standard conditions. In this regard, several constraints were established for data extraction. Thus, even if the study included diverse conditions in line with its experimental requirements, such as the presence of social, emotional or subliminal cues, we selected only the control conditions in which none of these special factors were present. That is, only morphosyntactic violations under neutral conditions entered the present analyses. Also, the LAN had to be obtained in visual reading conditions, avoiding the relatively noisy ERP signals usually obtained from auditory presentations. It was also required that the LAN obtained was distributed typically, i.e., clearly left frontal and with no other apparent fluctuations in the relevant interval.

Three of our published studies conformed with these constraints: Espuny et al. (2018), Hinchcliffe et al. (2020), and Martín-Loeches et al. (2012). In analyzing the neural origin of the LAN, we employed two distributed source models and a dipole model. The first distributed model was sLORETA (standardized low-resolution brain electromagnetic tomography; Pascual-Marqui, 2002), a standardization of the unweighted minimum norm estimate algorithm (Hämäläinen & Ilmoniemi, 1994). The second distributed algorithm was CLARA (classical LORETA analysis recursively applied; Scherg et al., 2022), which is an iterative approach of the classical LORETA (Pascual-Marqui et al., 1994). In each iteration, CLARA reduces the source space, yielding a better-defined spatial weighting, improving over the usually smeared signal of other distributed algorithms (Jordanov et al., 2014). Both algorithms have the advantage that no user-predefined locations are assumed. In order to improve the precision of the source localization, in a subsequent step the solutions suggested by the distributed models were exported to and analyzed through BESA dipole modeling (brain electrical source analysis; Scherg & Von Cramon, 1985). With this procedure, we aimed to overcome the inherent smearing in distributed models, objectively comparing and determining which exact locations are the best candidates for the neural origins of the LAN within the solutions provided by distributed algorithms.

The use of two formally different distributed algorithms (sLORETA and CLARA) is intended as a comprehensive approach offering the advantage of enhancing the reliability of the solutions provided in case of concurrence. In case of disagreement, the different solutions should be deemed and further checked throughout the BESA methodology in order to reach suitable and acceptable conclusions.

## MATERIALS AND METHODS

### Participants

The pooled participant sample of the three studies used here consisted of 76 graduate students (57 females, 19 males) with a mean age of 24.56 years (*SD* = 6.53). All participants had self-reported normal or corrected-to-normal vision, no history of neural or cognitive disorders, were not on psychiatric medications, and were all right-handed (*M* = 81.7%, range = 30–100), according to the Edinburgh Handedness Inventory (Oldfield, 1971). All studies were conducted in accordance with the Declaration of Helsinki and had been approved by the ethics committee of the Complutense University of Madrid.

### Stimuli

The ERPs for the present reanalysis were taken from three studies (Espuny et al., 2018; Hinchcliffe et al., 2020; Martín-Loeches et al., 2012), as described in the following. The stimulus material for the ERPs taken from Martín-Loeches et al. (2012) consisted of 120 out (of a total of 360) Spanish sentences following the structure: [Det]-[N]-[Adj]-[V] (determiner-noun-adjective-verb). The critical word (CW) was the adjective, which was of neutral emotional valence. Since the original study had aimed at investigating the effects of the emotional valence of the CW on syntactic processing, the 120 sentences selected for the present study were interspersed with 240 other sentences in which the CW was either emotionally positive or negative. Half of the 120 selected sentences contained a morphosyntactic (number) violation in the CW.

The language material from Espuny et al. (2018) consisted of 120 (out of a total of 360) Spanish sentences also following the structure of [Det]-[N]-[Adj]-[V] where the (neutral) adjective was the CW. Since the original study had investigated the effects of the emotional valence of a word on the syntactic processing of a subsequent sentence, the 120 sentences used here were interspersed with 240 other sentences with the same characteristics but preceded by an emotionally valenced word (see procedures below); the 120 sentences used here were preceded by a neutral word. Half of these sentences contained a morphosyntactic (number or gender, equiprobably) violation in the CW.

From the study of Hinchcliffe et al. (2020), ERPs to the correct and the morphosyntactically incorrect versions of a pool of 300 sentences were used. They included both short sentences ([Det]-[N]-[Adj]-[V]-[Complement]) and long sentences that could exhibit either one of three possible structures: ([Det]-[N]-[Adj]-[V]-[Preposition]-[Complement]), ([Det]-[N]-[Adj]-[V]-[Det + Preposition contracted]-[Complement]), or ([Det]-[N]-[Adj]-[V]-[Det]-[Complement]). The complements could be nouns, verbs, or words of other categories. Morphosyntactic violations were located in the adjective and in the verb for the short and long sentences, respectively. Mismatches in the adjective consisted in either gender or number violations, whereas in the verb they were always number violations. The original study investigated the effects of the mere presence of another person close to the participant on language processing in the semantic and syntactic domains. We selected only the ERP recordings in which the participant was alone. The selected sentences used here were interspersed with sentences containing either a semantic violation or a different structure.

For more details on the psycholinguistic variables, such as length of the CWs, frequency, etc., we refer to the original studies. Overall, the sentences selected here fulfilled two criteria. First, even if the corresponding study included other conditions (in line with its experimental requirements, as described in the paragraphs above), we selected only the control (neutral, standard) conditions. Second, and most importantly, the CWs had elicited a typical left frontally distributed negativity (LAN) with no other apparent fluctuations in the relevant interval when contrasting incorrect vs. the correct sentences (see below). Hence, only two conditions were contrasted here: morphosyntactically incorrect vs. correct sentences.

### Procedures

In all three experiments, participants performed sentence correctness judgements, that is, they were required to categorize each sentence as syntactically correct or incorrect by pressing one of two buttons with their index fingers). In the study of Martín-Loeches et al. (2012), all sentences were preceded by a cross in the middle of the screen for 500 ms and were presented word-by-word for 300 ms each, with a stimulus onset asynchrony (SOA) of 600 ms. One second of blank screen after the offset of the last word, a question mark appeared for 1 s, prompting the participant’s response. All stimuli were presented in white at the center of a black computer screen.

The presentation in Espuny et al. (2018) adhered to the same procedure with two exceptions. For one, the cross preceding the first word of each sentence lasted 300 ms and was followed by a 700-ms blank interval. Second, each sentence was preceded by a neutral word (in the sentences discarded for the present study these words were emotionally valenced), presented for 600 ms before the onset of the cross preceding the sentence (i.e., 1.6 s before the onset of the first word of the sentence).

In the study of Hinchcliffe et al. (2020), the procedure was as in Martín-Loeches et al. (2012) except that word duration was 200 rather than 300 ms and the SOAs were 500 rather than 600 ms. In this experiment, participants performed the task either alone or in the presence of a confederate, in different blocks; only the data of the alone block were used here. For more details on the procedures, we again refer to the original studies.

### EEG Recordings and ERP Analyses

EEG was recorded according to the 10/20 International System, from 27 scalp electrodes in Martín-Loeches et al. (2012) and 59 electrodes in Espuny et al. (2018) and Hinchcliffe et al. (2020). On-line references were the left mastoids (M1). Vertical and horizontal eye movements were monitored. The impedances of all electrodes were kept below 5 KΩ. Raw data were sampled at 250 Hz and recorded with a band-pass from .01 to 40 Hz in Martín-Loeches et al. (2012), and .01 to 100 Hz in Espuny et al. (2018) and Hinchcliffe et al. (2020).

Offline, the data were recalculated to a common average reference of both mastoids and filtered with a .01–30 Hz band-pass in all three studies. The continuous signal was segmented into 1-s epochs (1.2 s in Hinchcliffe et al., 2020), starting 200 ms before the onsets of the CWs; the 200 ms prestimulus intervals were used as baselines. Ocular correction was applied as well as artifact rejection. Incorrectly classified trials were removed. Further details on recordings and previous analyses can be seen in the original studies.

**Figure 1.**
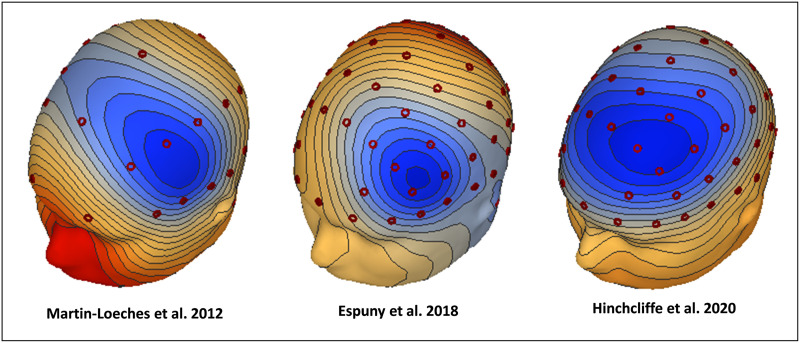
Scalp topographies of the LAN. ERPs to incorrect minus correct CWs in the three studies included in the present analyses. All maps scaled to ±1.5 µV.

Contrasting ERPs to syntactically incorrect and correct sentences yielded statistically significant LAN effects in all three studies. In both Martín-Loeches et al. (2012) and Espuny et al. (2018) the window at which this component was maximal and analyzed was 350–450 ms while in Hinchcliffe et al. (2020) it was 400–500 ms. In the three experiments, the LAN exhibited its typical left anterior topography for the conditions explored (Figure 1). Slight differences in topography could also be accounted, however, which seems to be typical of the variability intrinsic to this component (see Introduction). Even though, all three LANs represent the effects of the same psycholinguistic phenomenon, that is, the early effect of a morphosyntactic violation. Here we sought to elucidate whether there are common neural origins of these LAN effects and where they are localized.

### Source Analysis: The Present Study

Source analysis of the LAN was comprehensively approached in several steps. These are sketched in Table 1. First, with the aim of exploring the data in depth and without a priori definition of the sources, two distributed solution algorithms, sLORETA and CLARA, were applied as different but complementary methodologies. While sLORETA is a widely accepted algorithm used in previous analyses of the ELAN (e.g., Herrmann et al., 2011), CLARA reduces to some extent the spatial blurring common for most distributed algorithms, such as sLORETA. Its recursive approach allows it to differentiate neighboring sources instead of modeling a single, distributed source (Scherg et al., 2022). The solutions provided by the two distributed algorithms were then analyzed in a more detailed exploration using the equivalent dipole algorithm of BESA to specify the best candidate sources of the LAN. The BESA algorithm provides discrete solutions that can be delineated as precisely specified 3D coordinates. All the algorithms were applied as implemented in the BESA Research 7.1 software module (BESA GmbH, Gräfelfing, Germany).

**Table 1.** Steps in the source analyses procedures.

**A. Distributed source analyses**
1. sLORETA: individual-participant solutions in nAm/cm^3^ for correct and incorrect trials ERP separately, followed by cluster-based permutation testing of correctness effect (LAN)
2. CLARA: individual-participant solutions in nAm/cm^3^ for correct and incorrect trials ERP separately, followed by cluster-based permutation testing of correctness effect (LAN)
**B. BESA dipole analyses**
3. Find locations for equivalent dipoles in the mean correct-trial ERPs from distributed solutions (sLORETA and CLARA). Three locations were established.
4. Take the coordinates of the three dipoles in Step 3 for correct-trial ERP and insert them at the same location in all individual participants and let them rotate freely.
5. Take the individual solutions obtained from correct-trial ERP in Step 4, insert them with fixed location and orientation into the incorrect-trial ERP.
6. To the results in Step 5 add one of six dipoles in turn and calculate the Residual Variance (RV). These six dipoles were established according to the distributed solutions in steps 1 and 2.
7. Compare the RVs of the six solutions of Step 6 to determine the best location.
8. If the best location in Step 7 does not reduce RV to less than 20%, do the same as in 6 with plausible pairs of dipoles.

The analyses performed with sLORETA and CLARA followed very similar steps. To reduce solution spreading we applied CLARA with two iterations, and in both algorithms a constraint was applied to exclude the cerebellum as possible solution. In order to avoid distortions in the analyses, no constraint was imposed on subcortical solutions. However, since MEG and EEG signals are generated by postsynaptic potentials in the pyramidal neurons of the cerebral cortex (Lopes da Silva, 2013), subcortical solutions were disregarded as implausible (e.g., Guo et al., 2020; Paul-Jordanov et al., 2024). Regularization was set at 1% of the data variance to stabilize solutions and reduce noise sources. Following typical procedures, these distributed algorithms were applied to the average of the whole, 100-ms wide time window of interest for the LAN corresponding to each study (as described above) in order to attain subject-by-subject individual solutions. This also permits that differences in LAN latency between studies and in the number of electrodes are homogenized, yielding a unified, single sample that can be treated as a unitary group.

sLORETA and CLARA solutions for each participant were obtained for the correct and the incorrect (morphosyntactic violations) trials separately, which were thereafter submitted to cluster-based permutation testing using the BESA Statistics 2.1 software module (BESA GmbH, Gräfelfing, Germany) comparing voxel-by voxel (7-mm size) nAm/cm^3^ values for incorrect and correct conditions (LAN effects). Alpha was set to .05, two-tailed, and the number of permutations was always 1,000. The locations of the solutions, provided in Talairach coordinates, were further explored and defined using the MNI-to-Talairach coordinate converter provided by the BioImage Suite project (Yale University, https://bioimagesuiteweb.github.io/webapp/mni2tal.html), and the EBRAIN Human Brain Atlas (https://www.ebrains.eu/tools/human-brain-atlas).

Analyses with BESA consisted in two steps, again based on the same, 100-ms time segment corresponding to the LAN-window in each study, and in individual subject-by-subject solutions. First, based on the mean group solutions provided by sLORETA and CLARA algorithms for the correct trials only, three dipole positions appeared as sufficient to explain the variance in this condition (see below), stablished thereafter. They were used as a baseline for the syntactically incorrect conditions to which further dipoles were added in order to explain the additional, incorrectness-induced variance in the ERPs (the LAN effect), thereby minimizing the Residual Variance (RV, i.e., the variance unexplained by the dipoles). For the latter, single dipoles based on the solutions provided by the distributed algorithms, were added one by one to those three established for the correct condition. This way, we probed specific locations within the smeared distributed solutions, and the RV was checked individually per participant. These values were then submitted to repeated-measures ANOVA to better elucidate which single equivalent dipole best reduced RV.

## RESULTS

### Distributed Solutions

**Figure 2.**
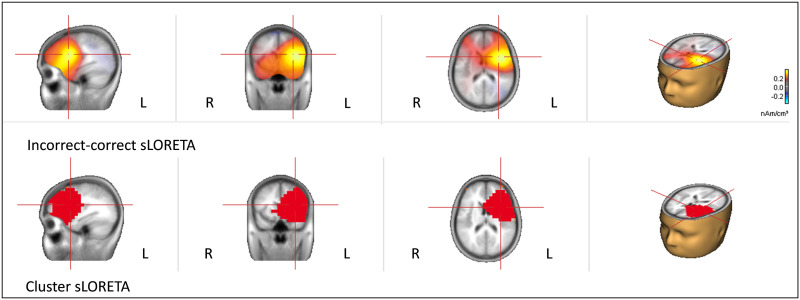
sLORETA results. Top: Differences between the sLORETA solutions obtained for the incorrect vs. the correct trials. Bottom: The significant cluster (*p* < 0.05) resulting from cluster-based permutation tests comparing the sLORETA solutions for incorrect vs. correct trials.

The results for the sLORETA algorithm can be seen in Figure 2. Only one cluster, mainly distributed over left frontal regions, yielded a significant effect of correctness (cluster value = 1980.09; *p* < 0.0001), with a maximum at Talairach coordinates −31.5, 11.07, 16.66 (maximum *t*-value = 455.9). This corresponds to the left deep FO contiguous to the anterior insula (LFO). The solution was quite broad and involved further adjacent regions, namely the left caudate nucleus, the left putamen, the left insula, and the left inferior frontal gyrus (LIFG).

**Figure 3.**
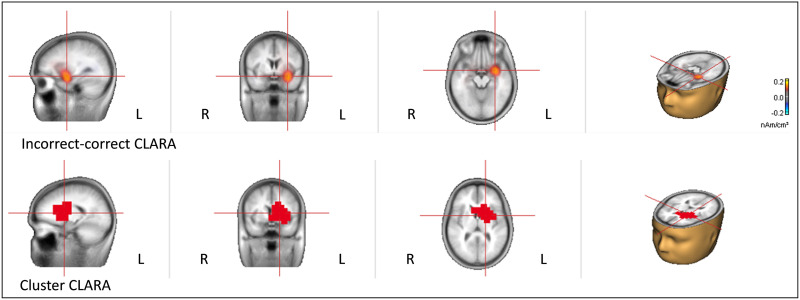
CLARA results. Top: Differences between the CLARA solutions obtained for the incorrect vs. the correct trials. Bottom: The cluster with significant permutation tests comparing the CLARA solutions for incorrect vs. correct trials.

The analyses performed with the CLARA algorithm can be seen in Figure 3. Again, only one cluster, primarily distributed within left frontal portions, was significant for correctness effect (cluster value = 330.75; *p* = 0.009), with a maximum at Talairach coordinates −17.5, 4.07, and 9.66 (maximum *t*-value = 380.8), which corresponds to the anterior limb of the left internal capsule. The cluster, which was more focused than the one obtained with sLORETA, nevertheless included also other neighboring regions, corresponding to the LFO, the left caudate nucleus, the left putamen, and the left insula.

Accordingly, two main results emerged as the maxima when localizing the neural source of the LAN in distributed sources analyses: the LFO according to sLORETA and the left basal ganglia (actually, internal capsule, adjacent to the caudate nucleus) according to CLARA. In both cases, the solutions also comprised other neighboring regions such as the left insula and the LIFG. An important consideration must be outlined here: the principal solution for CLARA (internal capsule) is not feasible for EEG or MEG data, as commented above, and therefore, this solution must be discarded. Accordingly, only cortical areas neighboring the main solution of CLARA are acceptable, that is, the LFO and the left insula.

### Discrete Dipoles (BESA)

Even if both distributed approaches coincided in suggesting the LFO as the best solution for the LAN effect (and possibly also the left Insula according to CLARA), their inherently distributed properties also point to other parts of the cerebral cortex. This prompted us to explore equivalent dipole solutions in different parts of the left frontal regions and to compare them in terms of RV as possibly better solution for the incorrect trials when added to the dipoles obtained from the correct conditions.

**Figure 4.**
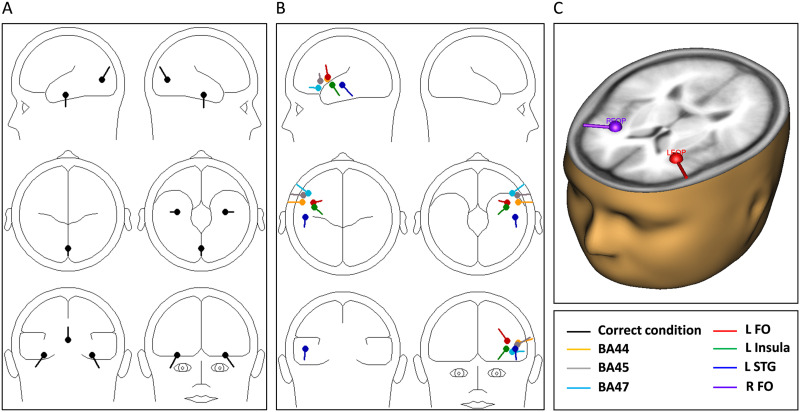
BESA results. (A) The dipole solutions yielding RV below 20% in the correct trial ERPs and used as baseline for the incorrect trial ERPs. (B) The six dipoles individually added to the solutions in A and tested in terms of RV in the incorrect trials. (C) The two final dipoles with RV below 20% in the incorrect trials, corresponding to the left and right FO.

Both sLORETA and CLARA solutions indicated that the correct trials in the LAN period can be explained by three neural sources, two of them located in bilateral basal portions of the temporal lobe, and the third one in the medial portions of the occipital lobe. By locating discrete dipoles in the center of these locations (Figure 4A) in each participant and allowing their orientations to move freely, the group mean RV obtained was 19.87%. This is below the 20% RV established as threshold for an acceptable RV value (Scherg & Von Cramon, 1986). These dipoles and their individual (participant-based) orientations based on correct trials were then submitted to the incorrect trials. This yielded a mean RV of 32.9%, which means that, as expected, the ERPs in this condition (including the LAN effect) cannot be sufficiently accounted for by the sources for the correct trials and need additional sources to reduce the RV to 20% or less. One by one, we then added up to six different individual dipoles to the data of the incorrect condition of each participant, placed at specific locations but allowing free orientations, and calculated the RV. The six left-hemisphere dipoles explored and their 3D coordinates, together with the main RV achieved after inserting each one, appear in Table 2 and are depicted in Figure 4B. The coordinates for the LFO are based on Friederici et al. (2000). The LIFG was subdivided in BA44, BA45 and BA47, areas that are usually considered as constituting Broca’s area. The coordinates of these three areas were obtained from their corresponding midpoints as provided by the BioImage Suite project (see above), as was done for the left insula. The dipole in the LSTG and its coordinates were taken from Herrmann et al. (2011) as it was their main solution for the ELAN and therefore it appeared of interest to be explored here.

**Table 2.** Mean coordinates and residual variances for six different single equivalent dipoles inserted into the individual incorrect-trial ERPs in addition to three dipoles taken from the solution for the correct-trial ERPs.

**Neural structure**	**Talairach coordinates**	**MNI coordinates**	**% RV after dipole**
L FO	−41 10 13	−43 12 12	23.83
L BA44	−57 10 11	−60 13 9	25.30
L BA45	−56 24 8	−58 27 6	25.06
L BA47	−51 31 −2	−52 35 −6	24.55
L Insula	−40 0 2	−42 4 −1	23.97
L STG	−52 16 2	−55 −15 −1	25.52

As can be seen in Table 2, the best dipole to explain the LAN effect seems to be located in the LFO, as there is the least RV after adding a source in this location (23.83%). However, the RV for a dipole in the left Insula is very similar while the values for the other dipoles are slightly larger. An ANOVA with factor Dipole Location (six levels) indicated significant differences between them (*F* = 14.46, *p* < .001). Post hoc, Bonferroni-corrected *p* < 0.05 pairwise comparisons were applied. Of interest, the dipole at LFO differed significantly from all other sources except for the left Insula, although the difference between the LFO and BA47 was only a trend (*p* = 0.056). The left Insula, in turn, did not differ significantly when compared with BA47, but it did so when compared with the other dipoles (except LFO). The LSTG, BA44, and BA45 did not differ significantly among them, while all did so from BA47, left Insula and LFO. In sum, even if all studied locations yielded relatively similar RV results, the analyses demonstrated significant differences among them, and the LFO seems to stand out from the others. Therefore, although not a completely clear-cut result, the LFO appears as the best solution for the LAN, as calculated through discrete dipole BESA analysis and supported statistically. The fact that this conclusion largely concurs with the maxima in the distributed solutions of sLORETA and (partially in) CLARA reinforces the confidence with which we can locate the LFO as the main neural source of the LAN.

Because the solution did not reach a reduction of the RV below 20%, it appeared necessary to add at least a second dipole. As adding any dipole at any given location would most probably reduce RV due to arithmetical reasons, we explored neuroanatomically plausible positions based on previous research (Scherg & von Cramon, 1985). Applying these constraints, two options appeared as the best candidates. One was the right FO, which is part of the solution outlined in Friederici et al. (2000) for the ELAN. In addition, the involvement in language processing of areas in the right hemisphere homologous to those in the left is a systematic finding (Duffau et al., 2014; Fedorenko et al., 2024), even if the left hemisphere is still dominant in this respect. Accordingly, we added a dipole in the homologous right-hemisphere coordinates of the LFO. Moreover, the spreading to medial frontal parts observed in the distributed solutions could indicate that cortices at both hemispheres might be contributing. The other option for a further dipole is the previously explored left STG, which is also a solution in Friederici et al. (2000). When the RFO dipole was added to the one in the LFO and both were allowed to orient freely, the mean RV was 19.17% (up from 23.83% for the LFO alone). When the LSTG was added, RV was 20.64%. The two values were significantly different from each other (*t* = 3.25, *p* < 0.001). In sum, a second dipole in the RFO added to the LFO appears as a better solution than a second dipole in the LSTG; in addition, the former combination reduces the RV to values below the conventional 20% threshold. Extra checking was performed by analyzing other possible pairs of dipoles. This again supported that the best solution appears to be LFO + RFO, as can be seen in the Supplementary Material; Supporting Information can be found at https://doi.org/10.1162/NOL.a.227. The two dipole locations in the LFO and RFO can be seen in Figure 4C.

## DISCUSSION

In pursuit of the neural origins of the LAN component of the ERP to morphosyntactic violations, the best candidate appears to be the left deep FO adjacent to the anterior insula (LFO). Both, the two distributed solutions analyses, as well as the dipole solutions concurred in supporting this assertion. A closely related solution could be the left Insula. Nevertheless, the latter appears to us as a less suitable candidate than the LFO. For one, the amount of variance explained by an Insula dipole is slightly lower than by the LFO dipole, the latter being also the main maximum solution in at least one of the distributed algorithms (sLORETA). Second, although the insula has been related to language processing, this was primarily for speech articulation and phonological processing, and only rarely in grammatical operations (Ardila et al., 2014). Indeed, the primary functions of the insula appear to be taste perception, interoception and emotion, and the integration of interoceptive and exteroceptive sensory processing (e.g., Zhang et al., 2024). In contrast, there is a long tradition and ample experimental evidence supporting the involvement of the LFO in syntax processes (e.g., Friederici, 2017; Friederici et al., 2003, 2006; Grodzinsky & Friederici, 2006; Heim et al., 2002; Meykadeh et al., 2021). Therefore, it seems that the most feasible neural origin of the LAN—or most of it—is the LFO.

At first sight, it may appear paradoxical that the putative origin of the LAN largely concurs with the source for the earlier component, the ELAN, hypothetically related but functionally dissociable (see Friederici, 2002, 2017). However, as mentioned in the Introduction, this dissociation is contested (Freunberger et al., 2022), as is even the reliability and validity of the ELAN itself (Fromont et al., 2016; Steinhauer & Drury, 2012). Indeed, the different latencies of ELAN and LAN might be the result of differences between languages and/or methodological issues (e.g., Molinaro et al., 2011). For instance, the dissociation of ELAN and LAN does not seem to hold in Spanish (Hinojosa et al., 2003; Molinaro et al., 2011), which is the language of the material used in the present analyses. In this regard, Bornkessel-Schlesewsky and Schlesewsky (2019) discussed that predictive coding models, which frame some ERP components as indicating prediction errors, might unify these components under a shared neurobiological mechanism, suggesting that ELAN and LAN might be better understood as part of a continuum of neural responses to syntactic anomalies rather than as wholly separable processes. In sum, the ELAN and LAN components may not be actually separable since both seem to reflect the activity of the deep FO, mainly of the left hemisphere, when a syntactic violation, for example, of a word category or of morphosyntax, is detected by the brain.

Taking these assumptions into consideration, we may consider a functional interpretation of the processes reflected by the LAN in the light of recent neuroscientific research. Such interpretation, however, should be considered with caution and as to some degree speculative, assuming results based on a technique with anatomical limitations (see below) and accepting that alternative interpretations might also be admissible. Following up on the interpretation by Bornkessel-Schlesewsky and Schlesewsky (2019), the component may stem from mechanisms related to prediction errors in relation to syntactic structures (see also Alekseeva et al., 2024). While the involvement of the FO in language processing seems undisputed (Woolnough et al., 2023), the type of language processes supported by this neural structure seems unclear. For instance, it has been found to be recruited for simple grammar, in contrast with the traditional Broca’s areas (i.e., BA44/45) processing more complex grammatical sequences (Friederici et al., 2006; Gallardo et al., 2023). However, Zaccarella and Friederici (2015) were unable to find a differential activation between syntactic phrases and word-list sequences within the FO. The latter appears primarily involved in motor planning (Mălîia et al., 2018), specifically of orofacial movements and articulation in speech (Amiez et al., 2023; Unger et al., 2023). On the other hand, the FO seems to be separate from the core language network as established in recent proposals (Fedorenko et al., 2024, 2020), pertaining instead to portions of the cerebral cortex more specifically involved—according to these proposals—in planning the motor movements needed for speech, a function in which even the adjacent Broca’s area as well as the insula would also be concerned (see also Hickok, 2022; Hickok & Poeppel, 2007). The Frontal Aslant Tract directly and ostensibly connects the FO with association motor areas, including the supplementary motor area, reinforcing the role of the FO in motor planning and sequencing (Briggs, Conner, et al., 2018). As the LAN is generated during language processing, its origin in the FO, according to the present results, might indicate that it is elicited by the detection of an interruption in an expected (predicted) utterance. Indeed, action and action perception straightforwardly contribute to language comprehension, by the generation of predictions. In this regard, speakers and language perceivers may use covert imitation and forward modeling to make predictions at phonological, syntactic and semantic levels, using these predictions to monitor the incoming information (Pickering & Garrod, 2013).

Given its anatomical position and functions, the predictions monitored by the FO during language comprehension might be primarily articulatory (morphological or morphophonological) in nature, even if largely dependent on syntactic principles. This is in line with interpretations (e.g., Bradley & Hestvik, 2010; Dikker et al., 2009; Lau et al., 2006) that the (E)LAN has to do more with mismatches of sensory expectancies, that is, of expected physical auditory parameters given a syntactic frame, than with ungrammaticality per se. Indeed, the FO is tightly connected to the anterior portions of the STG in BA22 through the uncinate fasciculus and the extreme capsule (e.g., Anwander et al., 2007; Friederici, 2017; Weiller et al., 2011). The anterior STG has not only been proposed as part of the origins of the ELAN (Friederici et al., 2000; Herrmann et al., 2009) but is also specialized in acoustic, phonetic feature encoding during speech perception (Mesgarani et al., 2014). This would occur even during reading, as the brain automatically converts written material into sounds or auditory representations through several pathways (i.e., spelling-to-sound, lexical, semantic conversion; e.g., Dehaene, 2009; Taylor et al., 2013), while the (E)LAN has been typically reported in both modalities. The interpretation is also in line with the fact that languages vary in the degree to which they make use of and rely on syntactic agreement marks (i.e., for gender, number, subject-verb, etc.), corresponding to the variability of the LAN across different languages (Friederici & Weissenborn, 2007; Molinaro et al., 2011). Thus, in Spanish, gender and number are marked mainly on final inflections, that is, on suffixes. Normally, masculine words end with an -o whereas feminine words end with an -a; for plural, an -s is the key rule. The same is also mostly the case for cues determining word category. Verbs, for instance, end in -ar, -er, or -ir in infinitive, or in recurrent suffixes to mark person, tense, mood, aspect (such as -é, -ría); adjectives (e.g., -oso, -al, etc.) and nouns (-ción, -ismo, -dad, etc.) have also distinctive suffixes (see Real Academia Española and Asociación de Academias de la Lengua Española, 2009). This means that specific sounds can be relatively reliably predicted when a word category or a morphosyntactic cue is expected. In sum, at least in Spanish (as well as in many languages in which the LAN has been studied), syntactic variables can be categorized mainly through distinctive sounds, that is, by acoustically distinct cues. When one of these cues does not match the one (or the few) expected, the FO triggers a resulting activation. This would probably arise before and even without needing subsequent syntactic processing per se in other, more specific language networks, since it would result from the interruption of a predicted sensory-motor sequence.

As reviewed in the Introduction, many studies report effects of extralinguistic factors affecting the LAN. This needs to be accounted for in light of the neural origins of the LAN proposed here. Indeed, some of these extralinguistic factors affecting the LAN comprise motor tasks performed during sentence processing (e.g., Casado et al., 2018), the execution of motor sequences being tightly related with the most outstanding functions of the FO (see above). However, the other types of extralinguistic non-motor variables appear more difficult to incorporate to this arguing, such as emotion, self-reference or social factors. Recent proposals suggest that when the brain is involved in complex and goal-directed tasks, metanetworks emerge, consisting of transient changes of relationship within and across neural networks (Herbet & Duffau, 2020). In brief, two or more brain functional, normally independent networks may work together in a context-sensitive manner, explaining apparently paradoxical results obtained with direct cortical stimulation during neurosurgical interventions, such as the responsiveness of Broca’s area to facial emotional expressions (e.g., Tate et al., 2014). It is therefore admissible that during a grammaticality judgment task the presence of other salient variables (emotional, social, etc.) might induce some degree of metanetworking and influence each other. Less speculatively, the direct connections of the FO with certain portions of the cerebral cortex might also account for most of those findings. In this regard, there is a direct and important connection of the FO with the anterior temporal pole (ATP, BA38; Briggs, Rahimi, et al., 2018). This part of the temporal lobe is actually associated with high-level semantic and socio-emotional information, autobiographical memory, social cognition, and face and object recognition, among others, acting as a hub integrating memory, emotion, and context (Herlin et al., 2021; Mesulam, 2023). It is also an important part of the Default Mode Network (DMN), critical for highest-level integration and essential, among other things, to construct a sense of self (e.g., Menon, 2023). Apparently, most of the nonsyntactical and extralinguistic factors influencing the LAN might do so through the connections between the FO and the ATP.

Our functional interpretations of the main processes underlying the LAN are provisional and in need of further support, and may apply only to situations in which grammatical sentence violations yield clearly left frontally distributed negativities, as analyzed here. However, several studies have reported negativities to morphosyntactic violations with centroparietal N400-typical topographies. Such effects have been found for various manipulations, such as gender or case violations (e.g., Choudhary et al., 2009; Frisch & Schlesewsky, 2001; Wicha et al., 2004), morphosyntactic reanalysis for word order variations (Bornkessel et al., 2004; Haupt et al., 2008) or object case ambiguities (Hopf et al., 1998). Remarkably, exactly the same violation that elicits a typical LAN turns into an N400-like distribution, in the same participants and in the same experimental session, by simply adding social or emotional information (Jiménez-Ortega et al., 2020; Martín-Loeches et al., 2012). In line with Bornkessel-Schlesewsky and Schlesewsky (2019), the N400 and the LAN might be members of a family of ERP components with similar functional characteristics, topographic differences reflecting divergences in the locus of prediction errors and model updating. Indeed, **s**everal factors may influence how a grammatical anomaly is handled. For instance, it has been proposed that the LAN/N400 scalp distribution could reflect a continuum where increasing amounts of lexical-semantic information involved add more N400-like effects in response to agreement errors (Molinaro et al., 2015). In line with this, it is our opinion that there are at least two possibilities. One is that the FO might still be involved in processing these morphosyntactic violations, but concurrently other regions may also participate, possibly modifying or modulating the topography of the negativity into a more central, N400-like distribution. But this would not necessarily be a genuine N400 effect. Alternatively, a genuine N400 effect may originate in other regions such as the anterior medial temporal lobe (e.g., Meyer et al., 2024), with no involvement of the FO. This might occur, for instance, when mainly lexico-semantic strategies or semantic information guide sentence processing. Our data do not permit to resolve this relevant question, deserving further investigation.

There are, of course, several limitations in the present study. Probably the most outstanding relate to inherent restraints in source analysis of scalp-recorded brain electrical activity. These include the inverse problem (as there are, in principle, infinite possible solutions), the low spatial resolution due to the distance between the scalp and the brain, or the distortion of the signal due to anatomical and other non-neural signals (e.g., Zorzos et al., 2021). This is an important limitation, which renders our results and interpretations rather tentative. Even though, our results seem robust enough to be endorsed, at least, as an acceptable solution. First, the result largely concurs with previous solutions in the literature, even if these were obtained with the limitations reviewed in the Introduction (e.g., currently outdated and less accurate algorithms, use of re-defined locations, reduced samples, etc.). They have been surpassed here, where not one, but several and more recently developed algorithms have been proven, and in a wide sample. The study by Herrmann et al. (2011) analyzing the ELAN with MEG also overcame at least part of these inconveniences, and found a different solution, in the anterior portions of the STG in BA22. As mentioned in the Introduction, it is possible that EEG and MEG measure different parts of the same phenomenon. Indeed, in our opinion, a contribution of the STG to the LAN is not incompatible with our results, while its functional interpretation could be largely similar, given the tight and apparent bidirectional connections between the anterior portions of the STG and the FO (Anwander et al., 2007; Friederici, 2017; Weiller et al., 2011) as well as its close anatomical proximity to the ATP.

## Conclusion

The LAN component of the ERP apparently emerges from the activation of the (left and right) FO when a predicted linguistic sequence is interrupted according to grammatical principles. Although it might not necessarily be a genuine syntax operation, it would be based on combinatorial restraints. Nevertheless, even if it is not syntax processing as such, its early and consistent involvement by morphosyntactic mismatches during sentence comprehension suggests that it likely reflects an essential, gateway process required to achieve thorough syntactic processing.

## Acknowledgments

This study was supported by Ministerio de Ciencia, Innovación y Universidades, Programa de ayudas a Proyectos de Generación de Conocimiento, Proyectos de Investigación no Orientada. Grant numbers: PID2021-123421NB-I00 & PID2021-124227NB-I00.

## Funding Information

Manuel Martin-Loeches, Ministerio de Ciencia, Innovación y Universidades (https://dx.doi.org/10.13039/501100004837), Award ID: PID2021-123421NB-I00. Laura Jimenez-Ortega and Pilar Casado, Ministerio de Ciencia e Innovación (https://dx.doi.org/10.13039/501100004837), Award ID: PID2021-124227NB-I00.

## Author Contributions

**Manuel Martin-Loeches:** Conceptualization; Data curation; Formal analysis; Funding acquisition; Methodology; Validation; Writing – original draft; Writing – review & editing. **Werner Sommer:** Conceptualization; Formal analysis; Methodology; Validation; Visualization; Writing – original draft; Writing – review & editing. **Laura Jiménez-Ortega:** Data curation; Funding acquisition; Validation; Writing – review & editing. **Javier Espuny:** Data curation; Validation; Writing – review & editing. **Miguel Rubianes:** Data curation; Validation; Writing – review & editing. **Pilar Casado:** Data curation; Validation; Writing – review & editing.

## Code and Data Availability Statement

Data -individual grand averages ERP for each participant and condition- from each of the three original studies are available from OSF public repository (https://osf.io/karns).

All the present analyses were performed using BESA (Brain Electrical Source Analysis), a third-party, commercial software (BESA GmbH, Gräfelfing, Germany).

## Supplementary Material



## References

[bib1] Alekseeva, M., Myachykov, A., Bermudez-Margaretto, B., & Shtyrov, Y. (2024). Morphosyntactic prediction in automatic neural processing of spoken language: EEG evidence. Brain Research, 1836, 148949. 10.1016/j.brainres.2024.148949, 38641266

[bib2] Aliko, A., Wang, B., Small, S. L., & Skipper, J. I. (2023). The entire brain, more or less is at work: ‘Language regions’ are artefacts of averaging. bioRxiv. 10.1101/2023.09.01.555886

[bib3] Amiez, C., Verstraete, C., Sallet, J., Hadj-Bouziane, F., Ben Hamed, S., Meguerditchian, A., Procyk, E., Wilson, C. R. E., Petrides, M., Sherwood, C. C., & Hopkins, W. D. (2023). The relevance of the unique anatomy of the human prefrontal operculum to the emergence of speech. Communication Biology, 6(1), 693. 10.1038/s42003-023-05066-9, 37407769 PMC10322890

[bib4] Anwander, A., Tittgemeyer, M., von Cramon, D. Y., Friederici, A. D., & Knösche, T. R. (2007). Connectivity-based parcellation of Broca's area. Cerebral Cortex, 17, 816–825. 10.1093/cercor/bhk034, 16707738

[bib5] Ardila, A., Bernal, B., & Rosselli, M. (2014). Participation of the insula in language revisited: A meta-analytic connectivity study. Journal of Neurolinguistics, 29, 31–41. 10.1016/j.jneuroling.2014.02.001

[bib6] Aurnhammer, C., Delogu, F., Brouwer, H., & Crocker, M. W. (2023). The P600 as a continuous index of integration effort. Psychophysiology, 60(9), e14302. 10.1111/psyp.14302, 37042061

[bib7] Bornkessel, I., McElree, B., Schlesewsky, M., & Friederici, A. D. (2004). Multi-dimensional contributions to garden path strength: Dissociating phrase structure from case marking. Journal of Memory and Language, 51(4), 495–522. 10.1016/j.jml.2004.06.011

[bib8] Bornkessel-Schlesewsky, I., & Schlesewsky, M. (2019). Toward a neurobiologically plausible model of language-related, negative event-related potentials. Frontiers in Psychology, 10, 298. 10.3389/fpsyg.2019.00298, 30846950 PMC6393377

[bib9] Bradley, E. D., & Hestvik, A. (2010). Testing the sensory hypothesis of the early left anterior negativity with auditory stimuli. LSA Annual Meeting Extended Abstracts. 10.3765/exabs.v0i0.524

[bib10] Briggs, R. G., Conner, A. K., Rahimi, M., Sali, G., Baker, C. M., Burks, J. D., Glenn, C. A., Battiste, J. D., & Sughrue, M. E. (2018). A connectomic atlas of the human cerebrum-chapter 14: Tractographic description of the frontal aslant tract. Operative Neurosurgery, 15(Suppl. 1), S444–S449. 10.1093/ons/opy268, 30260440 PMC6890526

[bib11] Briggs, R. G., Rahimi, M., Conner, A. K., Sali, G., Baker, C. M., Burks, J. D., Glenn, C. A., Battiste, J. D., & Sughrue, M. E. (2018). A connectomic atlas of the human cerebrum-chapter 15: Tractographic description of the uncinate fasciculus. Operative Neurosurgery, 15(Suppl. 1), S450–S455. 10.1093/ons/opy269, 30260439 PMC6890529

[bib12] Brouwer, H., Crocker, M. W., Venhuizen, N. J., & Hoeks, J. C. J. (2017). A Neurocomputational Model of the N400 and the P600 in Language Processing. Cognitive Science, 41(Suppl. 6), 1318–1352. 10.1111/cogs.12461, 28000963 PMC5484319

[bib13] Brouwer, H., Fitz, H., & Hoeks, J. C. J. (2012). Getting real about Semantic Illusions: Rethinking the functional role of the P600 in language comprehension. Brain Research, 1446, 127–143. 10.1016/j.brainres.2012.01.055, 22361114

[bib15] Casado, P., Martín-Loeches, M., León, I., Hernández-Gutiérrez, D., Espuny, J., Muñoz, F., Jiménez-Ortega, L., Fondevila, S., & de Vega, M. (2018). When syntax meets action: Brain potential evidence of overlapping between language and motor sequencing. Cortex, 100, 40–51. 10.1016/j.cortex.2017.11.002, 29212607

[bib16] Choudhary, K. K., Schlesewsky, M., Roehm, D., & Bornkessel-Schlesewsky, I. (2009). The N400 as a correlate of interpretively relevant linguistic rules: Evidence from Hindi. Neuropsychologia, 47, 3012–3022. 10.1016/j.neuropsychologia.2009.05.009, 19465035

[bib17] Ciaccio, L. A., Bürki, A., & Clahsen, H. (2023). Inter-individual variability in morphological processing: An ERP study on German plurals. Journal of Neurolinguistics, 67, 101138. 10.1016/j.jneuroling.2023.101138

[bib18] Dehaene, S. (2009). Reading in the brain: The science and evolution of a human invention. New York: Viking Penguin.

[bib19] Dikker, S., Rabagliati, H., & Pylkkänen, L. (2009). Sensitivity to syntax in visual cortex. Cognition, 110(3), 293–321. 10.1016/j.cognition.2008.09.008, 19121826 PMC2709501

[bib14] Duffau, H., Moritz-Gasser, S., & Mandonnet, E. (2014). A re-examination of neural basis of language processing: Proposal of a dynamic hodotopical model from data provided by brain stimulation mapping during picture naming. Brain and Language, 131, 1–10. 10.1016/j.bandl.2013.05.011, 23866901

[bib20] Espuny, J., Jiménez-Ortega, L., Hernández-Gutiérrez, D., Muñoz, F., Fondevila, S., Casado, P., & Martín-Loeches, M. (2018). Isolating the effects of word’s emotional valence on subsequent morphosyntactic processing: An event-related brain potentials study. Frontiers in Psychology, 9, 2291. 10.3389/fpsyg.2018.02291, 30519208 PMC6258783

[bib21] Fedorenko, E., Blank, I. A., Siegelman, M., & Mineroff, Z. (2020). Lack of selectivity for syntax relative to word meanings throughout the language network. Cognition, 203, 104348. 10.1016/j.cognition.2020.104348, 32569894 PMC7483589

[bib22] Fedorenko, E., Ivanova, A. A., & Regev, T. I. (2024). The language network as a natural kind within the broader landscape of the human brain. Nature Reviews Neuroscience, 25(5), 289–312. 10.1038/s41583-024-00802-4, 38609551 PMC13222024

[bib23] Fiebach, C. J., Schlesewsky, M., & Friederici, A. D. (2001). Syntactic working memory and the establishment of filler-gap dependencies: Insights from ERPs and fMRI. Journal of Psycholinguistic Research, 30(3), 321–338. 10.1023/a:1010447102554, 11523277

[bib24] Fodor, J. A. (1983). Modularity of mind. Cambridge, MA: MIT Press. 10.7551/mitpress/4737.001.0001

[bib25] Freunberger, D., Bylund, E., & Abrahamsson, N. (2022). Is it time to reconsider the ‘gold standard’ for nativelikeness in ERP studies on grammatical processing in a second language? A critical assessment based on qualitative individual differences. Applied Linguistics, 43(3), 433–452. 10.1093/applin/amab058

[bib32] Friederici, A. D. (1995). The time course of syntactic activation during language processing: A model based on neuropsychological and neurophysiological data. Brain and Language, 50(3), 259–281. 10.1006/brln.1995.1048, 7583190

[bib33] Friederici, A. D. (2002). Towards a neural basis of auditory sentence processing. Trends in Cognitive Sciences, 6(2), 78–84. 10.1016/S1364-6613(00)01839-8, 15866191

[bib26] Friederici, A. D. (2011). The brain basis of language processing: From structure to function. Physiological Review, 91(4), 1357–1392. 10.1152/physrev.00006.2011, 22013214

[bib27] Friederici, A. D. (2017). Language in our brain: The origins of a uniquely human capacity. MIT Press. 10.7551/mitpress/9780262036924.001.0001

[bib28] Friederici, A. D., Bahlmann, J., Heim, S., Schubotz, R. I., & Anwander, A. (2006). The brain differentiates human and non-human grammars: Functional localization and structural connectivity. Proceedings of the National Academy of Sciences, 103(7), 2458–2463. 10.1073/pnas.0509389103, 16461904 PMC1413709

[bib34] Friederici, A. D., & Kotz, S. A. (2003). The brain basis of syntactic processes: Functional imaging and lesion studies. NeuroImage, 20, S8–S17. 10.1016/j.neuroimage.2003.09.003, 14597292

[bib29] Friederici, A. D., Rüschemeyer, S.-A., Hahne, A., & Fiebach, C. J. (2003). The role of left inferior frontal and superior temporal cortex in sentence comprehension: Localizing syntactic and semantic processes. Cerebral Cortex, 13(2), 170–177. 10.1093/cercor/13.2.170, 12507948

[bib30] Friederici, A. D., von Cramon, D. Y., & Kotz, S. A. (1999). Language related brain potentials in patients with cortical and subcortical left hemisphere lesions. Brain, 122, 1033–1047. 10.1093/brain/122.6.1033, 10356057

[bib35] Friederici, A. D., Wang, Y., Herrmann, C. S., Maess, B., & Oertel, U. (2000). Localization of early syntactic processes in frontal and temporal cortical areas: A magnetoencephalographic study. Human Brain Mapping, 11(1), 1–11. 10.1002/1097-0193(200009)11:1<1::aid-hbm10>3.0.co;2-b, 10997849 PMC6872073

[bib31] Friederici, A. D., & Weissenborn, J. (2007). Mapping sentence form onto meaning: The syntax–semantic interface. Brain Research, 1146, 50–58. 10.1016/j.brainres.2006.08.038, 16956590

[bib36] Frisch, S., & Schlesewsky, M. (2001). The N400 reflects problems of thematic hierarchizing. NeuroReport, 12(15), 3391–3394. 10.1097/00001756-200110290-00048, 11711892

[bib37] Fromont, L. A., Royle, P., Perlitch, I., & Steinhauer, K. (2016). Re-evaluating the dynamics of phrase-structure processing using Event Related Potentials: The case of syntactic categories in French. International Journal of Psychophysiology, 108, 86. 10.1016/j.ijpsycho.2016.07.270

[bib38] Gallardo, G., Eichner, C., Sherwood, C. C., Hopkins, W. D., Anwander, A., & Friederici, A. D. (2023). Morphological evolution of language-relevant brain areas. PLoS Biology, 21(9), e3002266. 10.1371/journal.pbio.3002266, 37656748 PMC10501646

[bib39] Grevisse, D., Watorek, M., Heidlmayr, K., & Isel, F. (2023). Processing of complex morphosyntactic structures in French: ERP evidence from native speakers. Brain and Cognition, 171, 106062. 10.1016/j.bandc.2023.106062, 37473640

[bib40] Grodzinsky, Y., & Friederici, A. D. (2006). Neuroimaging of syntax and syntactic processing. Current Opinion in Neurobiology, 16(2), 240–246. 10.1016/j.conb.2006.03.007, 16563739

[bib41] Gross, J., Ioannides, A. A., Dammers, J., Maess, B., Friederici, A. D., & Müller-Gärtner, H.-W. (1998). Magnetic field tomography analysis of continuous speech. Brain Topography, 10(4), 273–281. 10.1023/a:1022223007231, 9672226

[bib42] Guo, Y., Bufacchi, R. J., Novembre, G., Kilintari, M., Moayedi, M., Hu, L., & Iannetti, G. D. (2020). Ultralow-frequency neural entrainment to pain. PLoS Biology, 18(4), e3000491. 10.1371/journal.pbio.3000491, 32282798 PMC7179945

[bib43] Hagoort, P. (2013). MUC (Memory, Unification, Control) and beyond. Frontiers in Psychology, 4, 416. 10.3389/fpsyg.2013.00416, 23874313 PMC3709422

[bib44] Hämäläinen, M. S., & Ilmoniemi, R. J. (1994). Interpreting magnetic fields of the brain: Minimum norm estimates. Medical & Biological Engineering & Computing, 32(1), 35–42. 10.1007/BF02512476, 8182960

[bib45] Haupt, F. S., Schlesewsky, M., Roehm, D., Friederici, A. D., & Bornkessel-Schlesewsky, I. (2008). The status of subject–object reanalyses in the language comprehension architecture. Journal of Memory and Language, 59(1), 54–96. 10.1016/j.jml.2008.02.003

[bib46] Hauser, M. D., Chomsky, N., & Fitch, W. T. (2002). The faculty of language: What is it, who has it, and how did it evolve? Science, 298(5598), 1569–1579. 10.1126/science.298.5598.1569, 12446899

[bib47] Heim, S., Opitz, B., & Friederici, A. D. (2002). Broca's area in the human brain is involved in the selection of grammatical gender for language production: Evidence from event-related functional magnetic resonance imaging. Neuroscience Letters, 328(2), 101–104. 10.1016/s0304-3940(02)00494-9, 12133565

[bib48] Herbet, G., & Duffau, H. (2020). Revisiting the functional anatomy of the human brain: Toward a meta-networking theory of cerebral functions. Physiological Review, 100(3), 1181–1228. 10.1152/physrev.00033.2019, 32078778

[bib49] Herlin, B., Navarro, V., & Dupont, S. (2021). The temporal pole: From anatomy to function—A literature appraisal. Journal of Chemical Neuroanatomy, 113, 101925. 10.1016/j.jchemneu.2021.101925, 33582250

[bib50] Herrmann, B., Maess, B., Hahne, A., Schröger, E., & Friederici, A. D. (2011). Syntactic and auditory spatial processing in the human temporal cortex: An MEG study. NeuroImage, 57(2), 624–633. 10.1016/j.neuroimage.2011.04.034, 21554964

[bib51] Herrmann, B., Maess, B., Hasting, A. S., & Friederici, A. D. (2009). Localization of the syntactic mismatch negativity in the temporal cortex: An MEG study. NeuroImage, 48(3), 590–600. 10.1016/j.neuroimage.2009.06.082, 19595773

[bib53] Hickok, G. (2022). The dual stream model of speech and language processing. Handbook of Clinical Neurology, 185, 57–69. 10.1016/b978-0-12-823384-9.00003-7, 35078610

[bib52] Hickok, G., & Poeppel, D. (2007). The cortical organization of speech processing. Nature Reviews Neuroscience, 8(5), 393–402. 10.1038/nrn2113, 17431404

[bib54] Hinchcliffe, C., Jimenez-Ortega, L., Muñoz, F., Hernández-Gutiérrez, D., Casado, P., Sánchez-García, J., & Martín-Loeches, M. (2020). Language comprehension in the social brain: Electrophysiological brain signals of social presence effects during syntactic and semantic sentence processing. Cortex, 130, 413–425. 10.1016/j.cortex.2020.03.029, 32540159

[bib55] Hinojosa, J. A., Martín-Loeches, M., Casado, P., Muñoz, F., & Rubia, F. J. (2003). Similarities and differences between phrase structure and morphosyntactic violations in Spanish: An event-related potentials study. Language & Cognitive Processes, 18(2), 113–142. 10.1080/01690960143000489

[bib56] Hopf, J.-M., Bayer, J., Bader, M., & Meng, M. (1998). Event-related brain potentials and case information in syntactic ambiguities. Journal of Cognitive Neuroscience, 10(2), 264–280. 10.1162/089892998562690, 9555111

[bib57] Jiménez-Ortega, L., Badaya, E., Hernández-Gutiérrez, D., Silvera, M., Espuny, J., Garcia, J. S., Fondevila, S., Muñoz, F. M., Casado, P., & Martín-Loeches, M. (2020). Effects of reader's facial expression on syntactic processing: A brain potential study. Brain Research, 1736, 146745. 10.1016/j.brainres.2020.146745, 32114058

[bib84] Jiménez-Ortega, L., Espuny, J., Herreros de Tejada, P., Vargas-Rivero, C., & Martín-Loeches, M. (2017). Subliminal emotional words impact syntactic processing: Evidence from performance and event-related brain potentials. Frontiers in Human Neuroscience, 11, 192. 10.3389/fnhum.2017.00192, 28487640 PMC5404140

[bib59] Jordanov, T., Hoechstetter, K., Berg, P., Paul-Jordanov, I., & Scherg, M. (2014). CLARA: Classical LORETA analysis recursively applied. In 20th Annual Meeting of Organization of Human Brain Mapping (OHBM). 2014 Jun 8–12. Hamburg, Germany.

[bib60] Kaan, E., Harris, A., Gibson, E., & Holcomb, P. (2000). The P600 as an index of syntactic integration difficulty. Language & Cognitive Processes, 15, 159–201. 10.1080/016909600386084

[bib61] King, J. W., & Kutas, M. (1995). Who did what and when? Using word- and clause-level ERPs to monitor working memory usage in reading. Journal of Cognitive Neuroscience, 7(3), 376–395. 10.1162/jocn.1995.7.3.376, 23961867

[bib62] Kluender, R., & Kutas, M. (1993). Bridging the gap: Evidence from ERPs on the processing of unbounded dependencies. Journal of Cognitive Neuroscience, 5(2), 196–214. 10.1162/jocn.1993.5.2.196, 23972154

[bib63] Knösche, T. R., Maess, B., & Friederici, A. D. (1999). Processing of syntactic information monitored by brain surface current density mapping based on MEG. Brain Topography, 12(2), 75–87. 10.1023/a:1023442426799, 10642007

[bib64] Lau, E., Stroud, C., Plesch, S., & Phillips, C. (2006). The role of structural prediction in rapid syntactic analysis. Brain and Language, 98(1), 74–88. 10.1016/j.bandl.2006.02.003, 16620944

[bib65] Lopes da Silva, F. (2013). EEG and MEG: Relevance to neuroscience. Neuron, 80(5), 1112–1128. 10.1016/j.neuron.2013.10.017, 24314724

[bib66] Mălîia, M.-D., Donos, C., Barborica, A., Popa, I., Ciurea, J., Cinatti, S., & Mîndruţă, I. (2018). Functional mapping and effective connectivity of the human operculum. Cortex, 109, 303–321. 10.1016/j.cortex.2018.08.024, 30414541

[bib67] Martín-Loeches, M., Fernández, A., Schacht, A., Sommer, W., Casado, P., Jiménez-Ortega, L., & Fondevila, S. (2012). The influence of emotional words on sentence processing: Electrophysiological and behavioral evidence. Neuropsychologia, 50(14), 3262–3272. 10.1016/j.neuropsychologia.2012.09.010, 22982604

[bib68] Martín-Loeches, M., Muñoz, F., Casado, P., Melcón, A., & Fernández-Frías, C. (2005). Are the anterior negativities to grammatical violations indexing working memory? Psychophysiology, 42(5), 508–519. 10.1111/j.1469-8986.2005.00308.x, 16176373

[bib69] Menon, V. (2023). 20 years of the default mode network: A review and synthesis. Neuron, 111(16), 2469–2487. 10.1016/j.neuron.2023.04.023, 37167968 PMC10524518

[bib70] Mesgarani, N., Cheung, C., Johnson, K., & Chang, E. F. (2014). Phonetic feature encoding in human superior temporal gyrus. Science, 343(6174), 1006–1010. 10.1126/science.1245994, 24482117 PMC4350233

[bib71] Mesulam, M. M. (2023). Temporopolar regions of the human brain. Brain, 146(1), 20–41. 10.1093/brain/awac339, 36331542 PMC10060717

[bib72] Meyer, P., Baeuchl, C., & Hoppstädter, M. (2024). Insights from simultaneous EEG-fMRI and patient data illuminate the role of the anterior medial temporal lobe in N400 generation. Neuropsychologia, 193, 108762. 10.1016/j.neuropsychologia.2023.108762, 38142959

[bib73] Meykadeh, S., Golfam, A., Batouli, S. A. H., & Sommer, W. (2021). Overlapping but language-specific mechanisms in morphosyntactic processing in highly competent L2 acquired at school entry: fMRI evidence from an alternating language switching task. Frontiers in Human Neuroscience, 15, 728549. 10.3389/fnhum.2021.728549, 34899211 PMC8663636

[bib74] Molinaro, N., Barber, H. A., & Carreiras, M. (2011). Grammatical agreement processing in reading: ERP findings and future directions. Cortex, 47(8), 908–930. 10.1016/j.cortex.2011.02.019, 21458791

[bib75] Molinaro, N., Barber, H. A., Caffarra, S., & Carreiras, M. (2015). On the left anterior negativity (LAN): The case of morphosyntactic agreement: A reply to Tanner et al. Cortex, 66, 156–159. 10.1016/j.cortex.2014.06.009, 25017646

[bib76] Oldfield, R. C. (1971). The assessment and analysis of handedness: The Edinburgh inventory. Neuropsychologia, 9(1), 97–113. 10.1016/0028-3932(71)90067-4, 5146491

[bib77] Osterhout, L., & Holcomb, P. (1992). Event-related brain potentials elicited by syntactic anomaly. Journal of Memory & Language, 31(6), 785–806. 10.1016/0749-596X(92)90039-Z

[bib78] Palolahti, M., Leino, S., Jokela, M., Kopra, K., & Paavilainen, P. (2005). Event-related potentials suggest early interaction between syntax and semantics during on-line sentence comprehension. Neuroscience Letters, 384(3), 222–227. 10.1016/j.neulet.2005.04.076, 15894426

[bib80] Pascual-Marqui, R. D. (2002). Standardized low-resolution brain electromagnetic tomography (sLORETA): Technical details. Methods and Findings in Experimental and Clinical Pharmacology, 24(Suppl. D), 5–12. 12575463

[bib79] Pascual-Marqui, R. D., Michel, C. M., & Lehmann, D. (1994). Low resolution electromagnetic tomography: A new method for localizing electrical activity in the brain. International Journal of Psychophysiolgy, 18(1), 49–65. 10.1016/0167-8760(84)90014-x, 7876038

[bib81] Paul-Jordanov, I., Hoechstetter, K., Bornfleth, H., Waelkens, A., Rusiniak, M., Cho, J.-H., Spangler, R., & Scherg, M. (2024). BESA research tutorial 4: Distributed source imaging. BESA Research Tutorial, 157–189. https://www.besa.de/wp-content/uploads/2024/02/BESA-Research-7.1-Tutorial.pdf.

[bib82] Pickering, M. J., & Garrod, S. (2013). An integrated theory of language production and comprehension. Behavioral and Brain Sciences, 36(4), 329–347. 10.1017/S0140525X12001495, 23789620

[bib83] Pulvermüller, F., & Shtyrov, Y. (2003). Automatic processing of grammar in the human brain as revealed by the mismatch negativity. NeuroImage, 20(1), 159–172. 10.1016/s1053-8119(03)00261-1, 14527578

[bib85] Real Academia Española and Asociación de Academias de la Lengua Española. (2009). Nueva Gramática de la Lengua Española. Madrid: Espasa Libros.

[bib86] Rubianes, R., Drijvers, L., Muñoz, F., Jiménez-Ortega, L., Almeida-Rivera, T., Sánchez-García, J., Fondevila, S., Casado, P., & Martín-Loeches, M. (2024). The self-reference effect can modulate language syntactic processing even without explicit awareness: An Electroencephalography study. Journal of Cognitive Neuroscience, 36(3), 460–474. 10.1162/jocn_a_02104, 38165746

[bib89] Scherg, M., Schulz, R., Berg, P., Cho, J.-H., Bornfleth, H., Kural, M. A., Woermann, F. G., Bien, C. G., & Beniczky, S. (2022). Relative source power: A novel method for localizing epileptiform EEG discharges. Clinical Neurophysiology, 133, 9–19. 10.1016/j.clinph.2021.10.005, 34788717

[bib87] Scherg, M., & Von Cramon, D. (1985). Two bilateral sources of the late AEP as identified by a spatio-temporal dipole model. Electroencephalography and Clinical Neurophysiology, 62(1), 32–44. 10.1016/0168-5597(85)90033-4, 2578376

[bib88] Scherg, M., & Von Cramon, D. (1986). Evoked dipole source potentials of the human auditory cortex. Electroencephalography and Clinical Neurophysiology, 65(5), 344–360. 10.1016/0168-5597(86)90014-6, 2427326

[bib90] Steinhauer, K., & Connolly, J.-F. (2008). Event-related potentials in the study of language. In B. Stemmer & H. A. Whitaker (Eds.), Handbook of the neuroscience of language (pp. 91–104). Elsevier.

[bib91] Steinhauer, K., & Drury, J. E. (2012). On the early left-anterior negativity (ELAN) in syntax studies. Brain and Language, 120(2), 135–162. 10.1016/j.bandl.2011.07.001, 21924483

[bib92] Sun, Y., & Luo, X. (2024). A mapping-knowledge-domain analysis of ERP research on language processing. Frontiers in Human Neuroscience, 18, 1352753. 10.3389/fnhum.2024.1352753, 38933147 PMC11199875

[bib93] Swaab, T. Y., Ledoux, K., Camblin, C. C., & Boudewyn, M. A. (2011). Language-related ERP components. In E. S. Kappenman & S. J. Luck (Eds), The Oxford handbook of event-related potential components (pp 398–440). Oxford Academic. 10.1093/oxfordhb/9780195374148.013.0197

[bib94] Tanner, D., & Van Hell, J. G. (2014). ERPs reveal individual differences in morphosyntactic processing. Neuropsychologia, 56, 289–301. 10.1016/j.neuropsychologia.2014.02.002, 24530237

[bib95] Tate, M. C., Herbet, G., Moritz-Gasser, S., Tate, J. E., & Duffau, H. (2014). Probabilistic map of critical functional regions of the human cerebral cortex: Broca’s area revisited. Brain, 137, 2773–2782. 10.1093/brain/awu168, 24970097

[bib96] Taylor, J. S. H., Rastle, K., & Davis, M. H. (2013). Can cognitive models explain brain activation during word and pseudoword reading? A meta-analysis of 36 neuroimaging studies. Psychological Bulletin, 139(4), 766–791. 10.1037/a0030266, 23046391

[bib97] Townsend, D. J., & Bever, T. G. (2001). Sentence comprehension: The integration of habits and rules. MIT Press.

[bib98] Turco, D., & Houghton, C. (2022). Bayesian modeling of language-evoked event-related potentials. arXiv. 10.48550/arXiv.2207.03392

[bib99] Unger, N., Haeck, M., Eickhoff, S. B., Camilleri, J. A., Dickscheid, T., Mohlberg, H., Bludau, S., Caspers, S., & Amunts, K. (2023). Cytoarchitectonic mapping of the human frontal operculum—New correlates for a variety of brain functions. Frontiers in Human Neuroscience, 17, 1087026. 10.3389/fnhum.2023.1087026, 37448625 PMC10336231

[bib100] Vieitez, L., Padrón, I., Díaz-Lago, M., de Dios-Flores, I., & Fraga, I. (2024). Unpleasant words can affect the detection of morphosyntactic errors: An ERP study on individual differences. Psychophysiology, 61(12), e14663. 10.1111/psyp.14663, 39086024 PMC11579219

[bib101] Weiller, C., Bormann, T., Saur, D., Musso, M., & Rijntjes, M. (2011). How the ventral pathway got lost: And what its recovery might mean. Brain and Language, 118(1–2), 29–39. 10.1016/j.bandl.2011.01.005, 21429571

[bib102] Wicha, N. Y. Y., Moreno, E. M., & Kutas, M. (2004). Anticipating words and their gender: An event-related brain potential study of semantic integration, gender expectancy, and gender agreement in Spanish sentence reading. Journal of Cognitive Neuroscience, 16(17), 1272–1288. 10.1162/0898929041920487, 15453979 PMC3380438

[bib103] Woolnough, O., Donos, C., Murphy, E., Rollo, P. S., Roccaforte, Z. J., Dehaene, S., & Tandon, N. (2023). Spatiotemporally distributed frontotemporal networks for sentence reading. Proceeding of the National Academy of Sciences, 120(17), e2300252120. 10.1073/pnas.2300252120, 37068244 PMC10151604

[bib104] Zaccarella, E., & Friederici, A. D. (2015). Merge in the human brain: A sub-region based functional investigation in the left pars opercularis. Frontiers in Psychology, 6, 1818. 10.3389/fpsyg.2015.01818, 26640453 PMC4661288

[bib105] Zhang, R., Deng, H., & Xiao, X. (2024). The insular cortex: An interface between sensation, emotion and cognition. Neuroscience Bulletin, 40(11), 1763–1773. 10.1007/s12264-024-01211-4, 38722464 PMC11607240

[bib106] Zorzos, I., Kakkos, I., Ventouras, E. M., & Matsopoulos, G. K. (2021). Advances in electrical source imaging: A review of the current approaches, applications and challenges. Signals, 2(3), 378–391. 10.3390/signals2030024

