# Supplementary material for: Gateway to Syntax: On the Neural Origins of the Left Anterior Negativity and Their Functional Implications
Source: Neurobiol Lang (Camb). 2026 Mar 26;7:NOL.a.227. doi: 10.1162/NOL.a.227 (PMC13065097; doi:10.1162/NOL.a.227)
Supplement: Supplementary file 1 [file nol-07-227-s001.pdf]

SUPLEMENTARY MATERIAL

As an extra checking, we wanted to explore the RV values obtained for other possible pairings, combining the LFO dipole with each of the other locations in the IFG and the Insula suggested by the distributed algorithms. The results can be seen in Table A below. An ANOVA (factor: Location of the dipole) indicated that the values differed among them (F=12,87; p<.001). The post-hoc comparisons with Bonferroni correction showed that LFO + LBA44, LFO + LBA45, LFO + LBA47, and LFO + L Insula combinations do not differ significantly between them. LFO + RFO differs significantly with all the others except with LFO + LSTG (a comparison that was significant in the main analyses but not here because of the Bonferroni correction), whereas LFO + LSTG differs significantly only with LFO + LBA44 and LFO + LB45. Overall, these results suggest that the best solution is LFO + RFO, which in addition is the only combination reducing RV to below 20%.

**Dipole pair**	**% RV after dipole pair**
L FO + L BA44	21.50
L FO + L BA45	21.41
L FO + L BA47	21.22
L FO + L Insula	21.25
L FO + L STG	20.64
L FO + R FOP	19.17

Table A. Residual variances for five different combinations of two single equivalent dipoles inserted into the individual incorrect-trial ERPs in addition to three dipoles taken from the solution for the correct-trial ERPs.
